# Lead Poisoning

**Published:** 1890-01

**Authors:** 

**Affiliations:** Berlin


### LEAD POISONING.

PROF. LITTEN, BERLIN.

A compositor suffered for many years from lead-intoxication in its lighter forms, when he began to suffer from a high grade of hyperæsthesia in the occipital region, in the muscles of the neck, shoulders, and upper extremities, combined with such an enormously increased reflex irritability in these muscles that merely touching the neck, a soft stroking, even a subjective turning of the head, sufficed to produce a lasting stiffness of the muscles with consequent very severe clonic twitchings, lasting for several minutes. With every turn of the head it was drawn downward to the shoulder of the same side and fixed in that position by the stony hardness of the muscles till the clonic twitchings began. No improvement from Sulphur bath and Kali-iod. This man of fifty-one years showed also a most interesting cutaneous affection: diffuse psoriasis over the whole body, existing since infancy, nearly disappearing at times and then rapidly spreading again over the whole body, so that hardly a spot remains free. The largest spots, with intensely red edges, and pale centres, covered with innumerable scales, are on the anterior surface of the lower extremities; on the posterior part of the neck and back, having the appearance of an old psoriasis; existing for years; and nobody, who saw the patient two weeks before, with a clean skin, could believe that the eruption is only two weeks old; beyond these were small fresh eruptions of the size of pinheads. It was a hereditary eruption dating back to infancy. A. M. C. Z., 72-89.

The pains of this compositor are well described by Litten, VIII, symptoms 2,583-2,590; and the tremors, 3,570-80; epileptiform spasms, 3,611, etc.; but as consciousness remained intact, they do not deserve the name of epilepsy in this case. 3,670, rigidity and tetanus; 3,796, excessive hyperæsthesia of the cutaneous nerves.

How that chronic psoriasis proves again the old psora theory of Hahnemann, going and coming for half a century and still uncured, notwithstanding all antiparasitic treatment. Did the long-continued lead poisoning have anything to do with that stubbornness of the cutaneous disease? What is meant by psora? In my lectures on the *Organon* I consider it a minor; a deficiency of vital power, a morbid disposition to disease, but even Hahnemann never satisfied me; let the philosophers of Homœopathy shed light upon this doctrine, though we know full well this hereditary or acquired disposition. S. L.

